# Metal-Organic Framework Reinforced Acrylic Polymer Marine Coatings

**DOI:** 10.3390/ma15010027

**Published:** 2021-12-21

**Authors:** Hwawon Seo, Inwon Lee, Vadahanambi Sridhar, Hyun Park

**Affiliations:** 1 Department of Naval Architecture and Ocean Engineering, Pusan National University, Busan 46241, Korea; ertyi0218@pusan.ac.kr (H.S.); inwon@pusan.ac.kr (I.L.); 2 Global Core Research Centre for Ships and Offshore Plants (GCRC-SOP), Pusan National University, Busan 46241, Korea

**Keywords:** metal organic frameworks, marine coatings, layered materials, microwave synthesis

## Abstract

Metal-organic frameworks (MOFs), a class of crystalline, porous, 3D materials synthesized by the linking of metal nodes and organic linkers are rapidly emerging as attractive materials in gas storage, electrodes in batteries, super-capacitors, sensors, water treatment, and medicine etc. However the utility of MOFs in coatings, especially in marine coatings, has not been thoroughly investigated. In this manuscript we report the first study on silver MOF (Ag-MOF) functionalized acrylic polymers for marine coatings. A simple and rapid microwave technique was used to synthesize a two-dimensional platelet structured Ag-MOF. Field tests on the MOF reinforced marine coatings exhibited an antifouling performance, which can be attributed to the inhibition of marine organisms to settle as evidenced by the anti-bacterial activity of Ag-MOFs. Our results indicate that MOF based coatings are highly promising candidates for marine coatings.

## 1. Introduction

The research being conducted on reticular structures that are comprised of the linkage of metal nodes and organic linkers to form crystalline, meso-porous, metal organic frameworks (MOFs), has significantly increased in the last three decades. MOFs have found extensive applications in areas such as catalysis [1], energy storage like lithium- [2] and sodium-ion batteries [3], super capacitors [4], electro-catalysts in water splitting [5], hydrogen generation from alcohols [6], hydrogen storage [7], water harvesting [8], CO_2_ sequestration [9], gas separation [10], luminescence [11], sensors [12] etc. MOFs are intrinsically modular in nature, which gives researchers excellent control over structure and porosity. The correct choice of an organic linker, based on the linker’s chelating ability, affinity to the metal node, strength of the metal–linker bond, and the symmetry of the moiety, has meant a wide range of MOFs ranging from 0-D quantum dots [13], 1-D nano rods [14], nano wires [15], nano belts [16], 2-D nano plates [17], nano sheets [18] and 3-D structures like clusters [19], flowers [20] etc., using different metals like Fe [21], Co [22], Zn [23], Ni [24], Mg [25], Zr [26] etc., have been obtained. However, when compared to these traditional metals, less attention has been focused on the synthesis of silver based MOF (Ag-MOF).

Metals such as Ag [27], Zn [28], and Cu [29] are very well known compounds that act as antibacterial agents. Though zinc is a nutritionally vital and essential compound for many enzyme actions and for the proper physiological functioning in human beings, persistent exposure to high doses of zinc does result in adverse health conditions like anemia and can cause pancreatic damage [30]. An attractive alternative to zinc is copper, which is used in agricultural pesticides as Cu nanoparticles can effectively control pests. However, even at low (5–20 mg Cu/plant) doses, there are adverse metabolic effects of Cu due to the generation of a reactive oxygen species (ROS) [31]. The utilization of copper in antifouling paints works on the principle of its release as Cu^+2^ ions, nano- and micron-sized particles. More than 95% of the released copper moieties depend on conditions like salinity, alkalinity, and organic matter content. The presence of complex ions in sea water eventually form insoluble Cu compounds like CuS, CuCl_2_, CuNO_3_ etc., and are released into the environment, which over time can accumulate to dangerously high toxic levels (>50–500 μg/L). Therefore, an attractive alternative to toxic zinc and copper is silver.

Silver is the one of the world’s earliest known materials to possess antimicrobial activity. The historian Herodotus, describes how the King of Persia, when going to war, took boiled water stored in flagons of silver as part of his provisions [32]. The first modern scientific description of antimicrobial activity involving silver was reported by Raulin in 1869, who reported the inhibiting activity of silver on the growth of *Aspergillus niger* [33]. The concept of oligodynamic activity has been behind the utility of silver in antimicrobial processes and products. One of the earliest and most studied was ‘Katadyn silver’, described as a spongy preparation of metallic silver, containing a small amount of added palladium or gold [34]. Despite disadvantages, such as its relatively high cost, silver is still an attractive option for antibacterial activity since it is active at low concentrations for long periods, is odorless and presents an extremely low toxicity risk to humans. However, the wide spread and prolonged use of silver nano-particles in antimicrobial applications is limited due to a decreased potency caused by aggregation (by Ostwald’s ripening), low stability, quick release of silver. One way to overcome these problems is to immobilize silver nanoparticles on a high surface area, using porous materials like silica, zeolite etc. One way of delaying the leaching of silver to lower levels without any adverse effect on its antibacterial properties is by ‘binding’ silver ions in Ag-MOFs [35,36,37].

Even though there are reports on the utility of Ag-MOFs, which have been prepared by using various organic linkers such as 1,2,4-triazole [38], benzimidazole [39], aminoterepthalic acid [40], pyridine dicarboxylic acid [41], phosphobenzoic acid [42], bipyridine [43] etc. as anti-bacterial agents, in all cases the anti-bacterial properties were tested in laboratory conditions using model Gram positive and Gram negative bacteria like *Escherichia coli* and *Staphylococcus aureus*, whereas there are no reports on ‘practical’ applications of Ag-MOF in real life conditions, especially in coatings where the utility of Ag-MOF is yet to be reported. In this manuscript we have investigated the utility of Ag-MOF in marine coatings. Microwave synthesis was utilized to prepare plate-like Ag-MOF, which was subsequently incorporated into acrylate based polymer resins to synthesize MOF reinforced marine coatings for the first time. Field tests of the MOF reinforced marine coatings in the Sea of Korea, near Pusan, exhibited an antifouling performance.

## 2. Experimental

### 2.1. Materials and Methods

Reagent grade chemicals silver acetate (CAS Number: 7664-93-9, ACS reagent grade, 98% purity), H2BTC (CAS Number: 7647-01-0, ACS reagent grade, 37 wt% in H_2_O), were purchased from Sigma-Aldrich, Korea, and were used as received. Microwave synthesis was carried out in a microwave oven manufactured by Daewoo, Korea (Model number: KR-B202WL, (Seoul, Korea) operated at 700 W and 2450 MHz frequency. Morphology of the Ag-MOF nanoplatelets and coatings was studied by Zeiss FEG-SEM Supra 25 (Seoul, Korea)(Field-emission scanning electron microscope operating at 10 kV). High resolution transmission electron microscopic images, high-angle annular dark-field (HAADF, Thermo Fisher Scientific Korea Ltd., Seoul, Korea) images and nano-scaled elemental maps were recorded on TALOS F200Xoperating at 200 kV (Thermo Fisher Scientific Korea Ltd., Seoul, Korea). Chemical moieties were studied by a Sigma Probe Thermo VG X-ray photoelectron spectrometer (Sigma probe, Thermo VG scientific, East Grinstead, UK). Deconvolution and curve fitting of XPS data was carried out using XPSPEAK 4.1 (Washington, DC, USA). Thermogravimetric analysis (TGA) was carried out on PerkinElmer, STA 6000 (PerkinElmer, Waltham, MA, USA). The elemental composition of Ag-MOF was tested by Vario-Micro Cube elemental analyzer (Langenselbold, Germany).

### 2.2. Synthesis of Ag-MOF Nanoplatelets

Though there are a number of techniques to synthesize MOFs, a microwave technique has been used in this study. 1.66 g of terephthalic acid (1 mmol) was dispersed in deionized water (10 mL), to which 1.66 g of silver acetate (1 mmol) was added and mixed thoroughly in an ultrasonicator for 30 min, after which the reactant was placed in a microwave oven and subjected to microwave irradiation for 120 s, which was subsequently allowed to cool naturally to room temperature, and the synthesized Ag-MOFs were separated from the reactant mixture by vacuum filtration, and dried in an oven.

### 2.3. Synthesis of Acrylic Polymer Resin

Acrylic polymer resin is based on methyl methacrylate (MMA), ethyl acrylate (EA), polyethylene glycol methacrylate (PEGMA), ethyl hexacrylate (EHA), trimethyl silylmethacrylate (TMSA) in propylene glycol monomethyl ether (PGM) and xylene solvent mixture (1:5) using azobis isobutyronitrile (AIBN) as an initiator and was synthesized according to our previous reports [44,45]. The synthesis was carried in a four-neck flask equipped with a condenser, thermometer, dropping funnel and stirrer at 95 °C at a mixing rate of 200 rpm for a reaction time totaling 4 h.

### 2.4. Preparation Method of of AgMOF/Polymer Resin Mixture 

A mixture of MOF and polymer resin was prepared using a disperser (Tokushu Kika, Homo Disper manufactured by Primix corporation (Tokyo, Japan). First, the measured amounts of resin and MOF powder were put in a container and Xylene was added as a solvent to facilitate the smooth mixing. For better particle dispersion and the exfoliation of the MOF nanosheets, zirconia balls used in ball milling were used. After maintaining the rotation speed at about 1200 rpm for 1 h, the milling ball was filtered out.

### 2.5. Immersion Test in Sea

First, the mixture of MOF/polymer was sprayed onto one side of PVC panel (10 cm × 30 cm) and left to dry for 1 week. The samples were fixed at 60–70 cm below sea level in Dadaepo (Busan) immersion test site for 2 weeks from 20 April 2021 to 4 May 2021. After installation, the extent of biofouling was observed visually using digital photographs. Image analysis was carried out by software that was developed in-house. Common organisms found in seawater at that time of year are: algae, bacteria, diatom, tube worm, barnacle, mussel, oyster, kelp, sea grass etc., besides animals like starfish, crabs, clams, anemones, fish etc.

## 3. Results and Discussion

**Figure 1:**
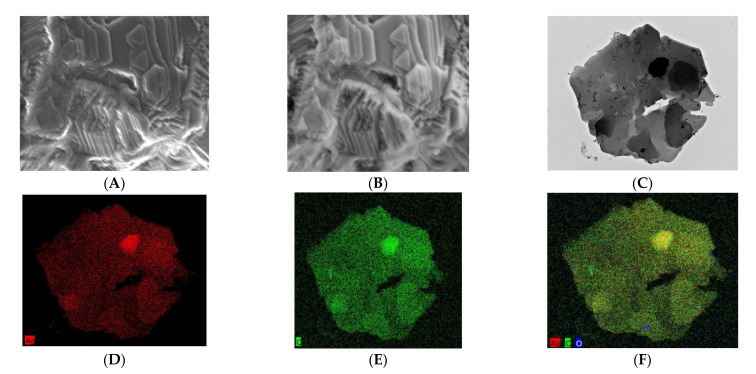
In-lens (**A**) and secondary ion emission (**B**) SEM micrographs of Ag-MOF nanoplatelets synthesized by microwave radiation; HRTEM (**C**) and its corresponding silver (**D**), carbon (**E**) and composite map (**F**). Scale bars are 1 µm in (**A**,**B**); 500 nm in (**C**–**F**), respectively.

The morphology and microstructure of Ag-MOFs synthesized by microwave technique was studied by in-lens and secondary ion emission SEM images exhibited in Figure 1A,B, respectively. In-lens image (Figure 1A) shows a highly agglomerated, platelet-like structure with well-defined and smooth surfaces with sharp truncated edges. Besides having sharp ‘geometric’ edges and sides, the majority of Ag-MOF nanoplatelets are hexagonal in shape with a minority being triangular or quadrilateral. Though there are thousands of papers on MOF, there are no reports on ‘secondary electron’ images of MOF nanostructures (Figure 1B). A back scattered, secondary electron image of Ag-MOF shows that a majority of MOF have a lateral dimension ranging from 400 to 500 nanometers, with a thickness in the range of 20–25 nm. A transmission electron microscopy (TEM) image of Ag-MOF, exhibited in Figure 1C, reflects the SEM data and shows that the MOF has an almost hexagonal structure with a smaller triangular MOF anchored on its surface. Though MOFs have been around for the past three decades, there is little understanding about the exact mechanism of its growth [46]. This lack of knowledge about the mechanism of MOF growth can be attributed to the existence of a variety of complex parameters such as compositional (molar ratios of starting materials, pH, solvent, etc.) and process parameters (reaction time, temperature, and pressure), which makes synthesis of MOF nanostructures more of an art than science. However, the general consensus about the growth mechanism of MOF micro crystals is based on the assumption that this takes place due to a particle–monomer attachment mechanism [47]. The higher the coordination number a metal has, the more complex three dimensional shapes an MOF can grow into. Under microwave radiation, H2BTC radicals are captured and coordinated by Ag ions, leading to the formation of a bridged ordered chain, which self-assembles into 2D Ag-MOF nanoplatelets [48]. Figure 1D shows the corresponding silver EDS map, which demonstrates that the silver moieties are well distributed throughout the MOF nanoplatelets, whereas the EDS map of carbon (Figure 1E) shows the concomitant presence of carbon with silver, which indicates there is good bonding between Ag^+^ ions and the H_2_BTC ions, which is also reflected in the composite Ag-C-O map shown in Figure 1F.

**Figure 2:**
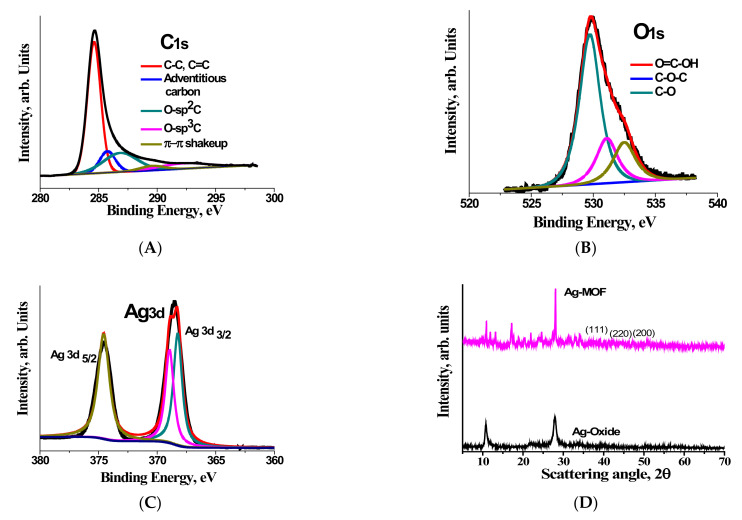
Deconvoluted C1 s (**A**), O 1s (**B**), Ag 3d (**C**) of Ag-MOF nanoplatelets. XRD patterns of Ag-MOF (**D**).

In order to further study the chemical structure of C, O and Ag moieties, X-ray photoelectron spectroscopy (XPS) was used. The deconvoluted C 1s XPS spectra exhibited in Figure 2A shows the typical 284.5 eV predominant peak assigned to C-C and C=C and three minor peaks: the 285.98 eV peak attributed to adventitious carbon [49]; the 286.9 eV peak corresponding to O-sp^2^-C configurations and the small minor peaks at 288.3 and 290.7 eV, which can be attributed to sp^3^ trigonal C-O bond and π-π shake up, respectively. The deconvoluted O 1s peak of Ag-MOF shown in Figure 2B indicates the presence of three major oxygen species differentiated by their binding energies (BE) namely the 529.7 to O=C-OH, 531.05 to C-O-C bonds and 532.4 eV to C-O of the carboxylate moiety of the organic ligand [50]. In order to study the chemical composition and structure of silver, high resolution XPS spectra of Ag 3d in the range of 360 to 380 eV were recorded and the deconvoluted spectra is plotted in Figure 2C, which shows two distinct peaks at centered at 374.6 eV and 368.5 eV corresponding to Ag 3d _5/2_ and Ag 3d _3/2_ and separated by a distance of 13.5 eV due to split spin-orbit components. The Ag 3d _3/2_ peak can be further deconvoluted into two peaks with almost equal areas centered at 368.3 and 369 eV [51]. Besides these, a small satellite peaks at 371 eV was also observed indicating that some metallic silver impurities (Ag(0)) also exist in our newly synthesized Ag-MOF platelets [52]. More information regarding the chemical moieties can be studied from FTIR spectra shown in Figure S1 of supplementary file. FTIR spectra of Ag-MOF is characterized by the presence of prominent peaks at 1606 and 1396 cm^−1^ corresponding to C=O bonds of benzene-carboxylic main group and vibrational mode of aromatic carbon group C-C bonds. Besides these, minor peaks of typical MOFs at 2086, 1586, 1410, 1166, 1041, 1013 cm^−1^ can also be seen [53]. The elemental composition of Ag-MOF as measured by elemental analysis shows the elemental composition of C: 56.39, H: 8.72, N: 0.0, O: 0.218%, which is in agreement with the elemental composition of other H2BTC based MOFs [54,55]. The thermal stability of Ag-MOF was tested by thermogravimetric analysis (TGA) under a nitrogen atmosphere (Figure S2 in supplementary file). TGA curve shows that Ag-MOF exhibits good stability and maintains an almost constant weight until 298 °C, after which there is a gradual weight loss due to the decomposition of the organic moiety, and at around 470 °C the weight stabilizes with a residual mass retention of 67.2% in line with other reports on TGA of H2BTC based MOF materials [53]. The XRD results of the Ag-MOF in Figure 1B shows Ag-MOF has a highly crystalline structure, which can be inferred from the presence of a sharp peak at 28.16 typical of Ag-organic moiety coordination compounds [56] with corresponding minor peaks at 10.9, 11.8, 13.04, 17.1 and 33.97 and the (1 1 1), (2 0 0), (2 2 0) peaks corresponding to 39.16, 43.84 and 65.31 to the face centered cubic crystalline planes of Ag-MOF nanoparticles [57].

**Figure 3:**
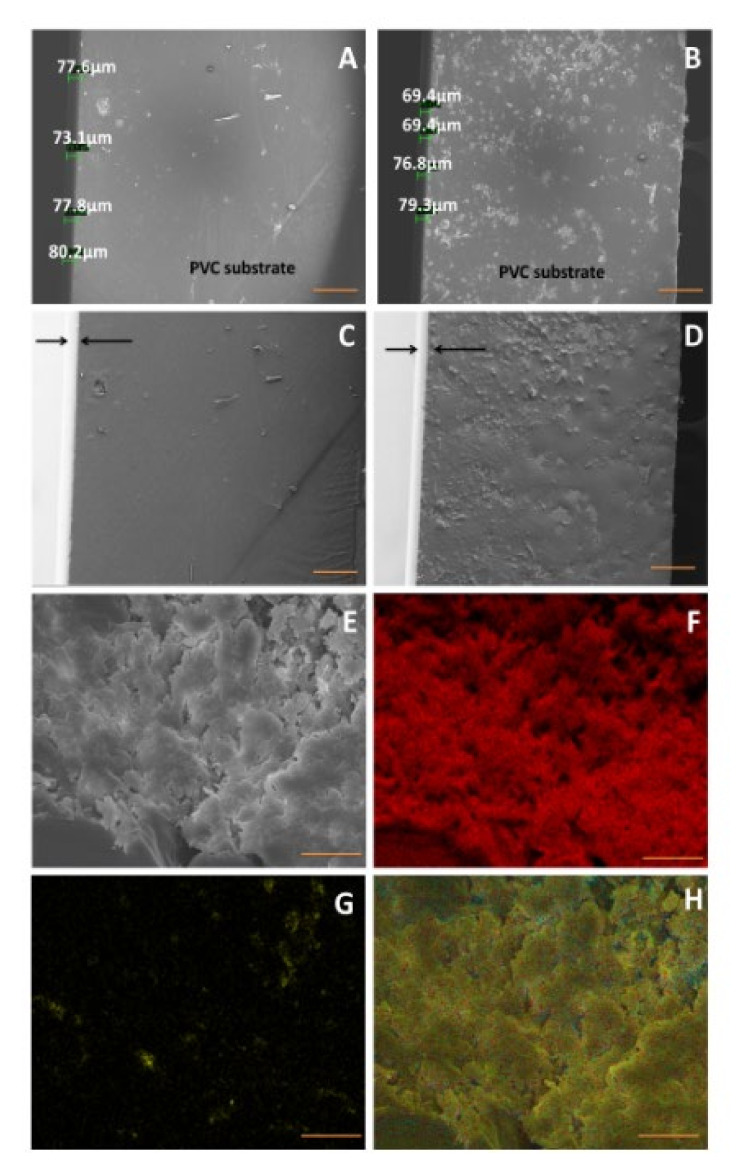
In-lens (**A**,**B**) and secondary electron (**C**,**D**) SEM morphologies of 1 wt% Ag-MOF reinforced acrylic coatings and pure resin coated on PVC substrate. Fracture surface of 1 wt% Ag-MOF reinforced acrylic coatings (**E**) and its corresponding carbon map (**F**), silver map (**G**) and composite map (**H**). Scale bar is 500 µm in (**A**–**D**); 20 µm in (**E**–**H**).

The morphology of 1 wt% Ag-MOF reinforced acrylic coatings and pure resin coated on PVC substrate as tested by SEM is shown in Figure 3A,B, respectively. The thickness of the films ranged from 69.4 to 79.3 µm in Ag-MOF reinforced acrylic coatings, whereas the pure resin coatings were 73.1 to 80.2 µm thick, which indicates reasonably uniform coatings are obtained. A clearer picture regarding the thickness of the films can be observed from the secondary electron image exhibited in Figure 3C,D wherein the acrylic marine coatings appear brighter when compared to PVC substrate, which appear substantially darker. Though there are innumerable reports on the study of coatings by regular in-lens SEM, this is the first report to have utilized a secondary electron image to study coatings. In order to study the state of dispersion of MOF in acrylic marine coatings, the SEM morphology of fracture surfaces of 1 wt% Ag-MOF reinforced acrylic coatings was examined (Figure 3E) and its corresponding carbon, silver and combined EDS maps are exhibited in Figure 3F–H, respectively. The silver EDS maps exhibits good dispersion and relatively good distribution of Ag-MOF nanoplatelets in acrylic resin coatings. The morphological studies above show that relatively uniform, micrometer thick coatings are obtained in our study.

**Figure 4:**
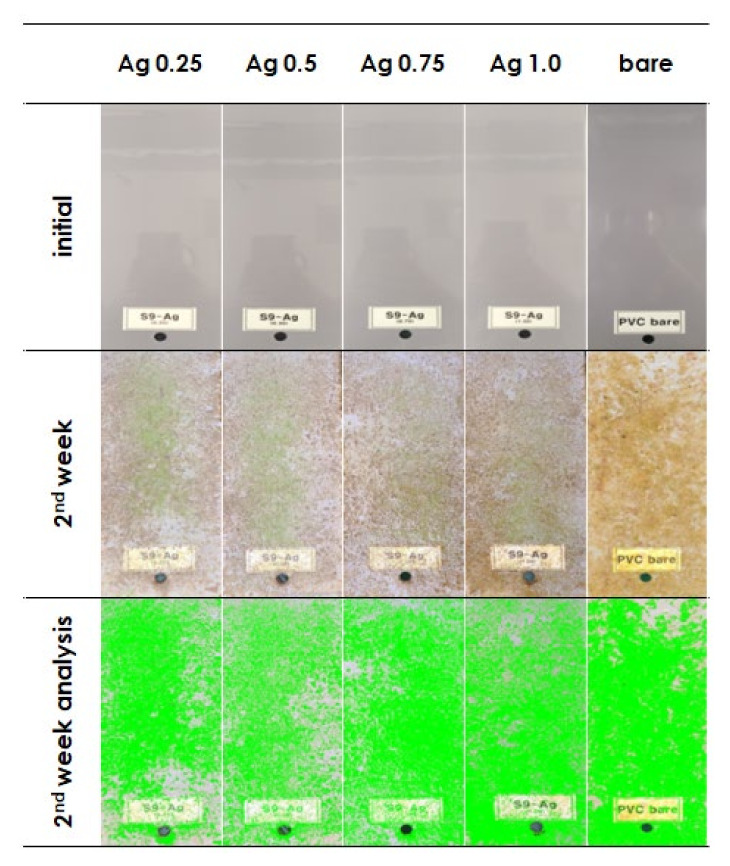
Images of Ag-MOF coated panels that were immersed for 2 weeks in the Korea sea (Dadaepo immersion test site, Busan).

Figure 4 shows the photographic images of panels coated with Ag-MOF reinforced acrylic coatings at concentrations of 0.25, 0.5, 0.75 and 1 wt% that were immersed in the Korea Sea for the first two weeks. In that first two weeks, the fouling of the surface was relatively less in the panel coated with the highest percentage (1 wt%) of Ag-MOF wherein the fouling in 0.25 wt% was relatively greater when compared to its counter parts. Total fouling area was measured by area analysis using image analysis software developed by our research group [57]. The total fouling area of bare showed 75.95% area covered by ‘organic growth’. This organic growth included plant based materials such as planktons and weeds and animal organisms such as mussels, barnacles etc. In the case of Ag-MOF reinforced acrylic coatings, the total fouling area was substantially lower at 54.77, 44.70, 66.26 and 63.01% for 0.25, 0.5, 0.75 and 1 wt% of Ag-MOF added into acrylic coatings, which indicates that the optimal loading of Ag-MOF for effective inhibition of marine fouling is 0.5 wt%.

The general belief is that under the prolonged immersion of coatings in seawater, an ion exchange reaction occurs wherein the silver ions of MOFs are gradually released from the Ag-MOG/acrylate coatings [58]. The efficacy of antifouling activity depends on the structure of the cell wall. Most living cells can be broadly divided into two categories: Gram positive and Gram negative. The cell wall of the two bacteria differs in that the peptidoglycan layer of the Gram positive bacteria is thicker than the Gram negative bacteria, and the Gram negative bacteria also have a unique outer membrane, which will provide additional protection [59]. However, when compared to traditional biocides, our Ag-MOF offers advantages such as: Bactericidal metal ions, since Ag is a known anti-microbial material and the inherent photo-catalytic antimicrobial activity due to the highest occupied molecular orbital–lowest unoccupied molecular orbital (HOMO−LUMO) gaps of silver, which is conducive for the formation of photo-generated reactive oxygen species (ROS) [60] organisms that give additional protection.

## 4. Conclusions

In summary, we report a fast and facile synthesis of Ag-MOF nanoplatelets within two minutes following simple microwave radiation. Morphological structural analysis by SEM reveals that our synthesized Ag-MOF has a plate-like structure and is 20–30 nm thick. HRTEM images show that the majority of Ag-MOFs are hexagonal in shape with minor triangular and quadrilateral MOFs anchored on the larger platelets. Elemental analysis by EDS maps show excellent and concomitant distribution of silver and carbon moieties in Ag-MOF, which was further analyzed by high resolution XPS. The utility of Ag-MOF in acrylate based marine coatings demonstrated that at all measured concentrations, the presence of Ag-MOF increased the antifouling capability.

## Data Availability

Not applicable.

## Supplementary Materials

The following are available online at https://www.mdpi.com/article/10.3390/ma15010027/s1, Figures S1 and S2: FTIR spectra and TGA of Ag-MOF, respectively.
