# Supplementary material for: Metal-Organic Framework Reinforced Acrylic Polymer Marine Coatings
Source: Materials (Basel). 2021 Dec 21;15(1):27. doi: 10.3390/ma15010027 (PMC8745788; doi:10.3390/ma15010027)
Supplement: Supplementary file 1 [file materials-15-00027-s001.zip › materials-1466918-supplementary.pdf]

Supplementary Material

Metal-Organic Framework Reinforced Acrylic Polymer Marine Coatings

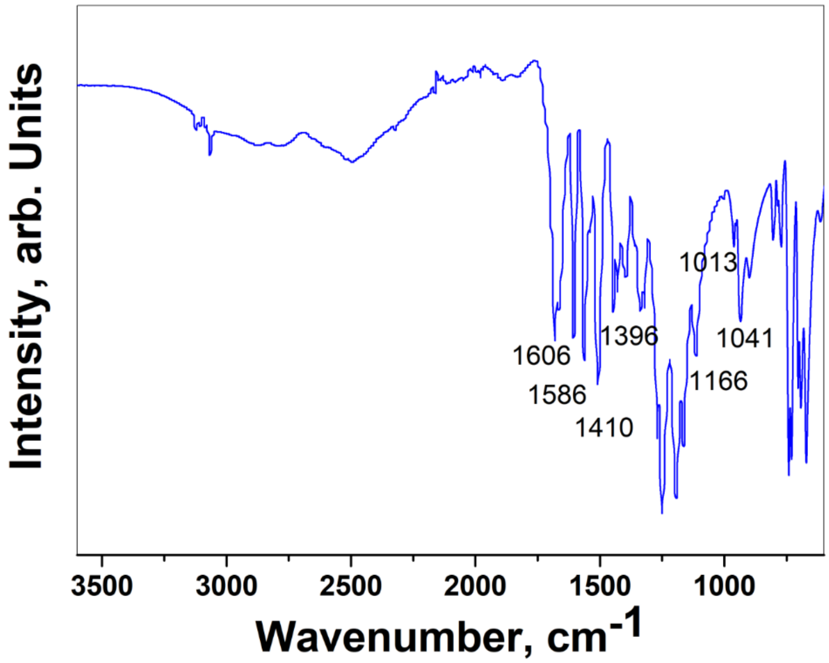

Figure S1. Fourier-transform infrared spectroscopy (FTIR) spectra of Ag-MOF.

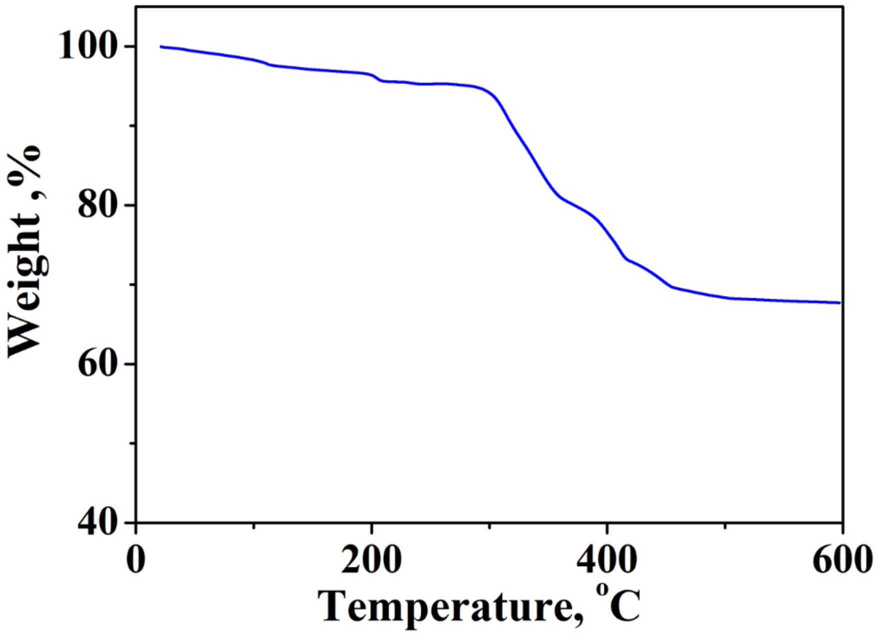

Figure S2. Thermogravimetric analysis (TGA) of Ag-MOF.
